# Improved Sleep, Memory, and Cellular Pathological Features of Tauopathy, Including the NLRP3 Inflammasome, after Chronic Administration of Trazodone in rTg4510 Mice

**DOI:** 10.1523/JNEUROSCI.2162-21.2022

**Published:** 2022-04-20

**Authors:** Paula de Oliveira, Claire Cella, Nicolas Locker, Kiran K. G. Ravindran, Agampodi Mendis, Keith Wafford, Gary Gilmour, Derk-Jan Dijk, Raphaelle Winsky-Sommerer

**Affiliations:** ^1^Surrey Sleep Research Centre, Department of Clinical and Experimental Medicine, Faculty of Health and Medical Sciences, University of Surrey, Guildford GU2 7XP, United Kingdom; ^2^Lilly Research Centre, Eli Lilly and Company, Windlesham GU20 6PH, United Kingdom; ^3^Department of Microbial and Cellular Sciences, School of Biosciences and Medicine, Faculty of Health and Medical Sciences, University of Surrey, Guildford GU2 7TE, United Kingdom; ^4^UK Dementia Research Institute Care Research and Technology Centre, Imperial College London, London W12 0BZ and University of Surrey, Guildford GU2 7XP, United Kingdom; ^5^Surrey Clinical Trials Unit, Department of Clinical and Experimental Medicine, Faculty of Health and Medical Sciences, University of Surrey, Guildford GU2 7XP, United Kingdom

**Keywords:** dementia, memory, NLRP3 inflammasome, sleep, tauopathy, trazodone

## Abstract

Several cellular pathways contribute to neurodegenerative tauopathy-related disorders. Microglial activation, a major component of neuroinflammation, is an early pathologic hallmark that correlates with cognitive decline, while the unfolded protein response (UPR) contributes to synaptic pathology. Sleep disturbances are prevalent in tauopathies and may also contribute to disease progression. Few studies have investigated whether manipulations of sleep influence cellular pathologic and behavioral features of tauopathy. We investigated whether trazodone, a licensed antidepressant with hypnotic efficacy in dementia, can reduce disease-related cellular pathways and improve memory and sleep in male rTg4510 mice with a tauopathy-like phenotype. In a 9 week dosing regimen, trazodone decreased microglial NLRP3 inflammasome expression and phosphorylated p38 mitogen-activated protein kinase levels, which correlated with the NLRP3 inflammasome, the UPR effector ATF4, and total tau levels. Trazodone reduced theta oscillations during rapid eye movement (REM) sleep and enhanced REM sleep duration. Olfactory memory transiently improved, and memory performance correlated with REM sleep duration and theta oscillations. These findings on the effects of trazodone on the NLRP3 inflammasome, the unfolded protein response and behavioral hallmarks of dementia warrant further studies on the therapeutic value of sleep-modulating compounds for tauopathies.

**SIGNIFICANCE STATEMENT** Dementia and associated behavioral symptoms such as memory loss and sleep disturbance are debilitating. Identifying treatments that alleviate symptoms and concurrently target cellular pathways contributing to disease progression is paramount for the patients and their caregivers. Here we show that a chronic treatment with trazodone, an antidepressant with positive effects on sleep, has beneficial effects on several cellular pathways contributing to neuroinflammation and tau pathology, in tauopathy-like rTg4510 mice. Trazodone also improved rapid eye movement (REM) sleep, the slowing of brain oscillations, and olfactory memory disturbances, which are all early symptoms observed in Alzheimer's disease. Thus, trazodone and compounds with REM sleep-promoting properties may represent a promising treatment approach to reduce the early symptoms of tauopathy and slow down disease progression.

## Introduction

Tauopathies are heterogeneous neurodegenerative disorders including Alzheimer's disease (AD) and frontotemporal dementia (FTD). These clinically presenting dementias are associated with pathologic deposits of abnormal tau protein (Kovacs, 2018). Current treatment strategies have primarily focused on AD and targeted amyloid peptide and abnormal tau. Other cellular features, such as neuroinflammation, play a crucial role in disease progression. Neuroinflammation is in part driven by the activation of resident microglia, which occurs early in AD and FTD (Leyns and Holtzman, 2017; Leng and Edison, 2021) and is related to early stages of tau pathophysiology (Sheffield et al., 2000; Laurent et al., 2018). Microglial activation positively correlates with tau burden in transgenic mice and tauopathy patients (Yoshiyama et al., 2007; Serrano-Pozo et al., 2011), and predicts cognitive decline and clinical severity in AD patients (Edison et al., 2008; Malpetti et al., 2020). The processes linking microglial activation to tau pathology and synaptic dysfunction remain poorly understood. Activated microglia recruit the p38 mitogen-activated protein kinase (MAPK) signaling pathway, which contributes to the spread of pathologic tau (Maphis et al., 2015). Recent findings indicate that the inhibition of the microglial Nod-like receptor protein 3 (NLRP3) inflammasome activation prevents tau pathophysiology and cognitive deficit in tau transgenic mice (Ising et al., 2019; Stancu et al., 2019).

Another putative contributing pathway is the unfolded protein response (UPR), triggered by an accumulation of misfolded proteins such as tau (Abisambra et al., 2013; Hughes and Mallucci, 2019). UPR activation correlates with tau burden in tauopathies (Nijholt et al., 2012) and the UPR PERK–eIF2α (eukaryotic translation initiation factor 2 α) pathway and its effector, the activating transcription factor 4 (ATF4), induce NLRP3 inflammasome activation (T. Li et al., 2020). Recently, it was reported that ATF4 and phosphorylated (p) tau^Thr212/Ser214^ levels were reduced in rTg4510 tauopathy mice by chronic administration of trazodone (40 mg/kg/d corresponding to 194 mg/d in humans (Halliday et al., 2017). Trazodone is a licensed antidepressant also commonly used off-label at lower than antidepressant doses (i.e., ∼50–200 mg/d) to treat sleep disturbances in older people (James and Mendelson, 2004). At these lower doses, trazodone also has beneficial effects on sleep duration and sleep efficiency in AD patients (McCleery and Sharpley, 2020). Sleep–wake disturbances are prominent symptoms even in the early stages of neurodegenerative diseases (Zhao et al., 2016; Sani et al., 2019; Winsky-Sommerer et al., 2019). These disturbances include excessive daytime sleepiness, an increased number of night awakenings, and a reduction in rapid eye movement sleep (REMS). In addition, a prominent slowing of electroencephalogram (EEG) activity in frontotemporal and parieto-occipital regions during REMS, and to a lesser extent during wakefulness, is a consistent finding in mild and advanced Alzheimer's disease, and has been associated with worsening of cognitive impairment (Musaeus et al., 2018; D'Atri et al., 2021). It has been estimated that sleep disturbances are responsible for 15% of AD cases (Bubu et al., 2017), and sleep disturbances are implicated in synaptic homeostasis and disease progression. In addition, chronic sleep restriction increased β-amyloid deposits and spread of tau accumulation in mouse models (Wang and Holtzman, 2020).

It is currently unknown whether a hypnotic dose of trazodone has simultaneous and positive effects on microglial activation and associated signaling pathways, as well as sleep and memory, in the context of tauopathy-related dementia. We therefore conducted a comprehensive assessment of the effects of chronic trazodone treatment on microglial activation, the NLRP3 inflammasome, the UPR, EEG sleep, and memory in rTg4510 mice, a model expressing the human P301L tau mutation linked to hereditary frontotemporal dementia. Here, we found that 9 week treatment with trazodone reduced the activation of several cellular components key to neuroinflammation and tau pathology, including microglial NLRP3 inflammasome expression, phosphorylated p38-MAPK, the UPR effector ATF4, and total tau levels. In parallel, trazodone also alleviated sleep disturbances and the decline of olfactory memory characteristic of tauopathy.

## Materials and Methods

### Mice.

Male rTg4510 (tet-o-TauP301L) bitransgenic mice were generated as previously described (Ramsden et al., 2005; Santacruz et al., 2005). Mice were licensed from the Mayo Clinic, bred on a mixed FVB/NCrl ^+^ 129S6/SvEvTa background by Taconic and delivered by ENVIGO. Age-matched wild-type (WT) mice, with the same genetic background, were used as control animals for immunoblotting assays. Mice were group housed (*n* = 2–3/cage) in standard home cages, except for animals used in the sleep experiment (cohort 1), which were housed individually after surgery and throughout the sleep study duration. Experimental rooms were kept under a constant 12 h light/dark cycle (∼35–40 lux at mid-level inside the cage), ambient temperature (21 ±1°C), and humidity (50 ± 15%). Mice were maintained on a food-restricted diet at no less than 85% of their initial free-feeding body weight. Food restriction started 1 week before the beginning of any intervention. Body weight was monitored throughout the studies. Water was provided *ad libitum*. All experiments were conducted in an Association for Assessment and Accreditation of Laboratory Animal Care International-accredited facility and conducted in accordance with the UK Animals (Scientific Procedures) Act 1986. Protocols and procedures were approved by the local Animal Welfare and Ethical Review Body (Eli Lilly and Company; University of Surrey).

### Experimental design.

**Figure 1.**
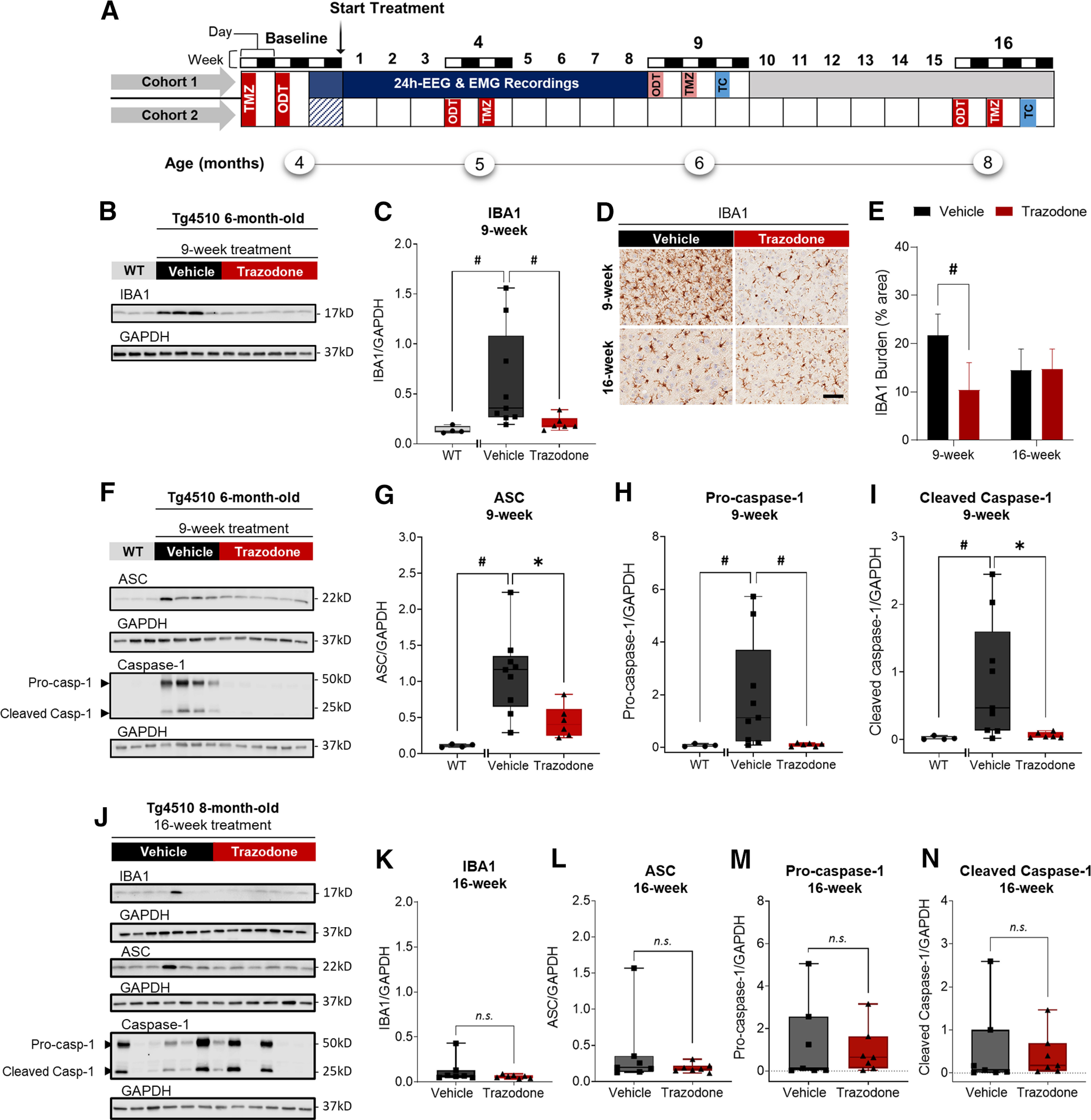
Trazodone inhibits microglial activation and NLRP3 inflammasome expression in the cortex of rTg4510 mice. ***A***, Experimental paradigm. Four-month-old rTg4510 mice were injected daily with trazodone (40 mg/kg) or vehicle over a period of 9 weeks (cohort 1) or 16 weeks (cohort 2). Age-matched WT samples were collected separately. Cohort 1 was used to assess treatment effect on sleep and the EEG, whereas cohort 2 was used to explore behavioral effects. Mice were kept under 12 h light/dark conditions (light bars: 12 h light phase; dark bars: 12 h dark phase). All behavioral testing was performed during the 12 h light phase. ODT, Odor discrimination test; TC, tissue collection; TMZ, T-maze test. ***B***, Representative immunoblots of microglial activation in cortical samples of WT and rTg4510 mice at 6 months old and after 9 weeks of treatment, respectively. ***C***, Protein quantification of IBA1 (WT vs vehicle: *U* = 0, *p* = 0.0028; vehicle vs trazodone: *U* = 5, *p* = 0.0076), in the cortex of 6-month-old untreated WT and rTg4510 mice after 9 weeks of trazodone treatment. ***D***, Representative cortical sections of trazodone- or vehicle-treated rTg4510 mice immunostained with IBA1 antibody (microglial activation); Scale bar, 50 µm. ***E***, IBA1 burden in the cortex of trazodone-treated rTg4510 mice after 9 weeks (*post hoc* analyses: *p* = 0.0029) and 16 weeks (*post hoc* analyses: *p* = 0.9392) of treatment. *n*(vehicle) = 9–10, *n*(trazodone) = 6–10. Data represented as LSMean ± 95% CI. Data analyzed using a general linear mixed model with repeated measures (SAS version 9.4). ***F***, Representative immunoblots of the main NLRP3 components in cortical samples of WT and rTg4510 mice at 6 months old and after 9 weeks of treatment, respectively. ***G–I***, Protein quantification of ASC (WT vs vehicle: *U* = 0, *p* = 0.0028; vehicle vs trazodone: *U* = *6, p* = 0.0120; ***G***), pro-caspase-1 (WT vs vehicle: *U* = 1, *p* = 0.0056; vehicle vs trazodone: *U* = 5, *p* = 0.0076; ***H***), and cleaved caspase-1 (WT vs vehicle: U = 1, *p* = 0.0056; vehicle vs trazodone: *U* = 7, *p* = 0.0176; ***I***) in the cortices of 6-month-old untreated WT and rTg4510 mice after 9 weeks of trazodone treatment. ***J***, Representative immunoblots of the IBA1 and NLRP3 inflammasome components in cortical samples of rTg4510 mice after 16 weeks of treatment. ***K–N***, Protein quantification of IBA1 (*U* = 17, *p* = 0.3829; ***K***), ASC (*U* = 18, *p* = 0.7308; ***L***), pro-caspase-1 (*U* = 20, *p* = 0.6200; ***M***), and cleaved caspase-1 (*U* = 18, *p* = 0.4557; ***N***) in the cortices of 8-month-old rTg4510 mice after 16 weeks of treatment. Immunoblot data were analyzed using the Mann–Whitney test (two tailed). Nine week treatment: *n*(WT) = 4, *n*(vehicle) = 9 or *n*(trazodone) = 6; 16 week treatment: *n*(vehicle) = 7 or *n*(trazodone) = 7; three to eight technical replicates per sample. Values shown in graphs are the band intensity of each protein divided by the intensity of GAPDH, with the results expressed as the respective median relative protein expression level ± minimum/maximum. ^n.s.^*p* > 0.05, *p < 0.05, #*p* < 0.01, $*p* < 0.001.

Experiments were conducted using two separated cohorts. In a first cohort (Fig. 1*A*), sleep and the EEG were monitored in 4-month-old male rTg4510 mice over an 8 week course of treatment. Behavioral testing was also performed after 8 weeks of treatment, and brain tissue was collected at the end of the experiment (i.e., after 9 weeks of treatment). To avoid confounding effects associated with EEG implantation, single-housing effects on behavior and reduce the number of invasive procedures, a second cohort of rTg4510 mice received chronic drug (or placebo) treatment and underwent behavioral tasks at baseline and after 4 and 16 weeks of treatment.

### Pharmacological treatment.

Mice were injected intraperitoneally with trazodone hydrochloride (40 mg/kg/d) or vehicle (0.25% methylcellulose) at Zeitgeber time 4 (ZT4) from 4 months of age. rTg4510 mice were treated for 9 weeks in the sleep experiment (up to 6 months old), or for 16 weeks (up to 8 months old) in the behavioral experiment. Treatment was randomized at the start of the experiment by cage. Experimenters were blind to treatment during clinical assessment and to scoring of the behavioral and sleep data.

### Surgical procedures for EEG/electromyogram sleep study.

Mice underwent in-house surgery at 3 months old (weight: 29.3 ± 0.4 g). Subjects were anesthetized (2% isoflurane in 100% oxygen, 0.1 mg/kg medetomidine HCl, s.c., injection) and surgically prepared for chronic EEG and electromyogram (EMG) recordings as previously described (Holton et al., 2020). The cranial implant consisted of five stainless steel screws for EEG recording (frontal electrode: 2 mm anterior to bregma and 2 mm left of the sagittal suture line; occipital electrode: 3 mm caudal from bregma and 3 mm right of the sagittal suture line; two stabilizing screws opposite to the frontal and occipital electrodes; one ground electrode over the cerebellum). Two Teflon-coated stainless steel wires were positioned in the nuchal trapezoid muscles for EMG recording. Atipamezole (0.5 mg/kg, s.c.) was administered to reverse the medetomidine. Carprofen (5 mg/kg, s.c.), a nonsteroidal anti-inflammatory drug, was administered preoperatively and postoperatively, and on the morning of the first postoperative day. One single dose of long-lasting prophylactic antibiotic treatment (cefovecin, 8 mg/kg, s.c.) was administered postoperatively.

### Polysomnography recordings.

Each individual recording chamber consisted of a standard home cage placed within a separate and shielded compartment. After a 15 d recovery from surgery, mice were connected to the acquisition setup and allowed to adjust for 7 d before starting recording over a continuous period of 8 weeks. Analog bipolar differential EEG and integrated EMG were amplified (10,000×) and digitized at 800 Hz with bandpass filters (EEG: 0.1–300 Hz; EMG: 10–100 Hz; Grass Corp.). Vigilance states [i.e., wakefulness, non-REM (NREM) sleep and REM sleep] were scored in each 10 s epoch based on a combination of salient EEG period and amplitude features and root mean square EMG using SCORE2004, an automated real-time sleep–wake monitoring system that was previously validated (Van Gelder et al., 1991; Mccarthy et al., 2017). EEG scoring was individually optimized to determine vigilance states, and visual signal inspection was performed to confirm vigilance state determination. Mice displaying high signal artifacts were excluded from further analysis (vehicle: *n* = 1; trazodone: *n* = 1). Time spent in wakefulness, NREM sleep (NREMS), and REM sleep were computed per hour, as well as by sleep bout count and average bout duration. Bout length was defined as one 10 s epoch episode or more of each specified arousal state. Sleep–wake data were represented as least square mean (LSMean)± 95% confidence interval (CI). The average of each week of data for each 12 h light and dark phase was used to reflect significant differences within the 24 h cycle.

### Spectral EEG analysis.

EEG power spectra for each 10 s epoch were computed using a fast Fourier transform. The spectrogram was then subdivided into the following bands: delta (0.5–3.9 Hz), theta (4.0–8.9 Hz), alpha (9.0–11.9 Hz), and beta (12.0–20.0 Hz). The sigma band (10-15Hz) was also computed for NREM sleep. The state-specific time series of EEG power in each band was computed for all EEG-defined epochs devoid of artifacts. Total power was computed over 0.1–30 Hz. EEG/EMG recordings were analyzed for a 24 h baseline and 8 weeks of treatment.

### Odor discrimination task.

Mice were previously habituated to the testing chambers for 30 min before each testing. The location of each mouse was randomized at the start of the study, and each mouse was positioned in the same testing box throughout. Olfactory memory was evaluated in a dark room using a clear open-top Plexiglas arena (40 × 40 × 30 cm) with a standardized tissue cartridge for odor delivery centered 2 cm from the bottom half of the arena (i.e., cartridge area). Testing was performed between light onset and ZT4, at baseline, and after 4 and 16 weeks of treatment. The olfactory discrimination task followed two steps: novel odor habituation and familiar odor discrimination. On the day preceding each assessment, mice were introduced to an appetitive odor using two to three Cheerios (∼6–9 g/mouse; Nestlé) for 5 min. Twenty-four hours after, subjects were presented with a novel and neutral odor in three consecutive trials (trials 1–3) to ensure a robust habituation to the novel stimulus. Each 2 min trial was separated by a 5 min intertrial interval (ITI). The novel odor used (e.g., 1:100 dilutions of pine, basil, or rosemary) was randomized between time points throughout the study. After a 30 min interodor interval, mice were then presented with the appetitive odor in a manner similar to the novel odor habituation stage (three consecutive trials of 2 min, with 5 min ITIs; trials 4–6). The time and frequency spent investigating the cartridge area during each trial were monitored using overhead infrared cameras (model VCV-3412P, Sanyo; from Tracksys Ltd.), which relayed data to a computerized video analysis software Ethovision XT version 12.0 (Noldus). As exposure to the novel odor and subsequent dishabituation by recognition of a familiar odor was the main probe of this olfactory memory task, the discrimination index was computed by subtracting the investigation time or frequency of the last novel odor habituation trial (trial 3) from that of the following first appetitive odor trial presentation (trial 4).

### Rewarded alternation T-maze test.

Spatial working memory was assessed at baseline, and after 4 and 16 weeks of treatment using a costume-made semiautomated T-maze (Apogee Engineering Analysis Solutions). Rewarded alternation T-maze test was performed between ZT0 and ZT4. Two sugar pellet dispensers were placed at the end of each choice arm. A rewarded alternation test was performed as previously described (Blackmore et al., 2017). First, during a sample phase, mice were forced to turn toward an arm containing a reward pellet. Forced left or right allocations during the sample phase were randomized with no more than three consecutive sample runs to the same side being permitted. During the rewarded alternation testing, mice could freely choose between the two arms, after a 5 s interval between phases. However, mice were only rewarded when visiting the arm not explored during the previous sample phase. Mice could explore the maze for up to 30 min or for a maximum of 20 trials. The total number of completed trials and the number of correct choices were recorded by a microcontroller (Arduino MEGA 2560, Arduino Software) using seven infrared beam breaks, while custom-made MATLAB (MathWorks) programs automatically controlled the maze doors and test procedure.

### Tissue collection.

Mice were terminally anesthetized with intraperitoneal pentobarbital (200 mg/kg in 5% glucose), and cardiac perfusion was performed with ice-cold PBS. The left hemisphere was collected, and the cortex was snap frozen in dry ice for immunoblotting purposes, while the right hemisphere samples were immersed and fixated in 10% neutral buffered formalin (prefilled vials; Leica Biosystems) and stored at room temperature until processed for immunohistochemistry (IHC) analyses.

### Immunoblotting.

Protein lysates were prepared in RIPA lysis buffer (catalog #R0278, Sigma-Aldrich) enriched with protease and phosphatase inhibitor cocktails (catalog # 4693132001 and catalog # 4906837001, Roche). Protein concentrations were determined using a Pierce BCA Protein Assay Kit (Thermo Fisher Scientific). An equivalent amount of protein from each sample was mixed with 3× Red Loading Buffer (catalog #7723, Cell Signaling Technology) with 1.25 m dithiothreitol (Cell Signaling Technology), and incubated at 95°C for 5 min before being separated in 4–15% gradient SDS-PAGE gels by electrophoresis (Mini-PROTEAN TGX, BIO-RAD). Samples were then transferred to 0.2 μm nitrocellulose membranes and blocked in either 5% nonfat dry milk or 5% bovine serum albumin in Tris-buffered saline supplemented with 0.025% Tween 20 for 1 h at room temperature. Membranes were immunoblotted overnight at 4°C with antibodies directed against ionized calcium-binding adaptor molecule 1 (IBA1; 1:500; 016–20001, Wako), total tau (tau5; 1:4000; catalog #AHB0042, Thermo Fisher Scientific), phospho-tau^Thr212, Ser214^ (AT100; 1:2000; catalog #MN1060; Thermo Fisher Scientific), apoptosis-associated speck-like protein containing a caspase-recruitment domain (ASC; 1:500; catalog #67824s, Cell Signaling Technology), caspase-1/pro-caspase-1 (1:500; catalog #sc-56036, Santa Cruz Biotechnology), phospho-p38 MAPK (Thr180/Tyr182; 1:1000; catalog #9216s, Cell Signaling Technology), p38 MAPK (1:1000; catalog #9212s, Cell Signaling Technology), ATF4 (1:1000; catalog #sc-390063, Santa Cruz Biotechnology), and glyceraldehyde-3-phosphate dehydrogenase (GAPDH; 1:4000; catalog #sc-32 233, Santa Cruz Biotechnology). Membranes were then treated with horseradish peroxidase-conjugated secondary antibodies for 1 h at room temperature. Immunoreactive bands were visualized using the Clarity Western ECL substrate solution (BIO-RAD) and the VILBER FUSION FX imaging system (Vilber Lourmat). Membranes were stripped once to allow reprobing of the loading control GADPH antibody. Signal intensity was analyzed using Image Studio Lite 5.2 software (LI-COR).

### Immunohistochemistry.

Right brain hemispheres were processed using the Tissue-Tek VIP processor (GMI) and embedded in paraffin wax (Tissue-Tek TEC, Sakura). Sagittal serial sections (6 µm) were collected using a rotatory microtome (catalog #HM 355S, Thermo Fisher Scientific). IHC was performed using primary antibodies directed against the IBA1 (1:4000; Wako) and phospho-tau^Ser202, Thr205^ (AT8; 1:4000; courtesy of Zoe Parton and Riazul Alam, Eli Lilly & Co.). Briefly, slides were subjected to heat-induced antigen retrieval in citrate buffer at 100°C for 20 min (catalog #TA-250-PM1X, Thermo Fisher Scientific) and then transferred to an automated stainer system (Lab Vision Autostainer 720 or 720N, Thermo Fisher Scientific). Slides were then incubated for 10 min in 0.3% hydrogen peroxide (1:100; Sigma Aldrich), followed by 30 min with normal goat serum (1:20; Vector Labs). Sections were subsequently incubated with primary antibodies. Biotinylated goat secondary antibody (1:200; DAKO) was then applied for 30 min, with the exception of bAT8-stained slides. Slides were incubated with avidin–biotin complex reagent for 30 min (Vectastain ABC Kit, catalog #PK-7100, Vector Labs) and treated with the peroxidase substrate 3,3′-diaminobenzidine (ImmPACT DAB Kit; catalog #SK-4105, Vector Labs). Brain sections were counterstained with hematoxylin for 5 min and coverslipped (ClearVue XYL Mountant, Thermo Fisher Scientific). Sections were digitized using the Scanscope XT slide scanner (Aperio) at 20× magnification. Imagescope software was used to delineate the cortex region. Staining was quantified using a positive pixel algorithm, as previously described (Blackmore et al., 2017).

### Statistical analyses.

Normality was tested for each dataset using the Shapiro–Wilk test and Q–Q normality plots. Statistical analyses of protein expression in the vehicle- and drug-treated groups were analyzed using the Mann–Whitney *U* test. Data were represented as individual values and median values (version 9.2.0, GraphPad Prism). Spearman's correlation analyses between protein expression markers were performed if the normality assumption was violated, based on normality test results (PROC CORR, SAS version 9.4). For repeated measures, such as IHC, sleep–wake, EEG, and behavioral data, data were analyzed using a general linear mixed-model approach (PROC MIXED for ANOVA) with treatment (trazodone vs vehicle group) and time (days or week) as categorical explanatory variables. *Post hoc* multiple pairwise comparisons (trazodone vs vehicle group) were assessed using the ESTIMATE option of PROC MIXED in SAS version 9.4 (SAS Institute). Corresponding baseline measures were used as covariates, except for IHC data. Because of the repeated nature of the data from the same subject, the within-subject correlation was accounted for using compound symmetry or variance–covariance structure. Data were reported using LSMean ± 95% CI in GraphPad Prism (version 9.2.0) or SigmaPlot version 14.0 (Systat Software). Because of reduced sample size, Kendall's tau correlation analyses between sleep and behavior parameters were performed if the normality assumption was violated, based on normality test results (PROC CORR, SAS version 9.4). Data were considered significant when *p* < 0.05 (two tailed).

## Results

### Chronic trazodone treatment reduced microglial activation and NLRP3 inflammasome expression in the cortex of rTg4510 mice

We first assessed the effects of 9 weeks of trazodone (40 mg/kg/d) administration (Fig. 1*A* ; cohorts 1 and 2) on IBA1, a marker of activated microglia, in the cortex of rTg4510 mice. Trazodone significantly reduced IBA1 expression after 9 weeks of treatment (Fig. 1*B*,*C*; *U* = 5, *p* = 0.0076, Mann–Whitney *U* test) in rTg4510 mice, in which IBA1 levels are upregulated compared to age-matched WT control mice (Fig. 1*C*; *U* = 0, *p* = 0.0028, Mann–Whitney *U* test). The reduction of IBA1 protein levels after 9 weeks of treatment was confirmed by immunohistochemistry (Fig. 1*D*,*E*: treatment: *F*_(1,33)_ = 5.81, *p* = 0.0217; treatment × week interaction: *F*_(1,33)_ = 6.30, *p* = 0.0172; general linear mixed model). No significant effect of trazodone on microglial activation was observed after 16 weeks of treatment (Fig. 1*E*). However, at this time point and age, IBA1 expression was lower in vehicle-treated rTg4510 mice (i.e., 8-month-old mice) compared with 6-month-old vehicle-treated mice.

We next assessed the expression of NLRP3 inflammasome components (i.e., ASC, pro-caspase-1, and cleaved caspase-1) in 6-month-old rTg4510 mice (Fig. 1*A*, cohort 1), when tau burden and microglial activation are pronounced (Santacruz et al., 2005; Wes et al., 2014), compared to age-matched WT controls. Protein levels of ASC (Fig. 1*F*,*G*; U = 5, *p* = 0.0028, Mann–Whitney *U* test), pro-caspase-1 (Fig. 1*F*,*H*; *U* = 1, *p* = 0.0056, Mann–Whitney *U* test), and cleaved caspase-1, a NLRP3 complex-free form of caspase-1 (Fig. 1*F*,*I*; *U* = 1, *p* = 0.0056, Mann–Whitney *U* test) were upregulated in rTg4510 mice, in accordance with previous reports in other mouse lines of tauopathy (Ising et al., 2019; Stancu et al., 2019), and in the brain or blood of patients with mild cognitive impairment, AD, and FTD (Saresella et al., 2016; Ising et al., 2019). Nine weeks of trazodone treatment reduced the expression of ASC compared with the parallel vehicle-treated mice (Fig. 1*G*; *U* = 6, *p* = 0.0120, Mann–Whitney *U* test) and normalized the levels of pro-caspase-1 (Fig. 1*H*; *U* = 5, *p* = 0.0076, Mann–Whitney *U* test) and cleaved caspase-1 (Fig. 1I; *U* = 7, *p* = 0.0176, Mann–Whitney *U* test), supporting a drug-induced inhibition of the NLRP3 inflammasome. After 16 weeks of treatment, no differences between the trazodone- and vehicle-treated groups were seen in IBA1 (Fig. 1*J*,*K*; *U* = 17, *p* = 0.3829, Mann–Whitney *U* test) or NLRP3 inflammasome expression (Fig. 1*J*,*L*; ASC: *U* = 18, *p* = 0.7308; Fig. 1*J*,*M*; pro-caspase-1: *U* = 20, *p* = 0.6200; Fig. 1*J*,*N*; cleaved caspase-1: *U* = 18, *p* = 0.4557, Mann–Whitney *U* test). These data show that trazodone reduces microglial activation and NLRP3 inflammasome expression after 9 weeks of treatment.

### Nine weeks of daily trazodone treatment reduced cortical protein levels of phosphorylated p38 MAPK, ATF4, and total tau in rTg4510 mice

**Figure 2.**
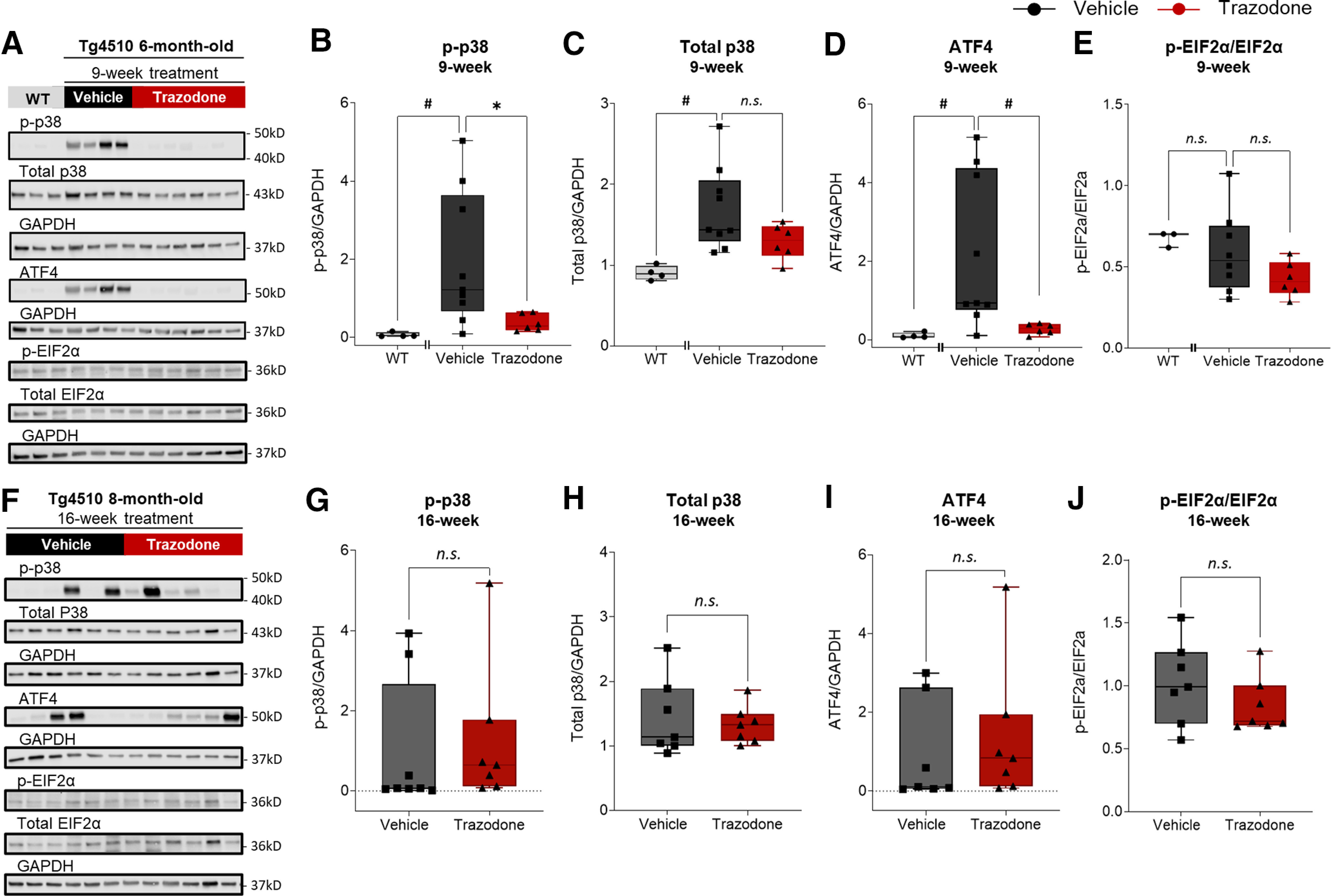
Cortical P38 MAPK and ATF4 levels are reduced in trazodone-treated rTg4510 mice. ***A***, Representative immunoblots of P38 MAPK, ATF4, and EIF2α in cortical samples of WT and rTg4510 mice after 9 weeks of treatment. Age-matched untreated WT samples were collected separately. ***B–D***, Protein levels of phosphorylated p38; p-p38; (WT vs vehicle: *U* = 1, *p* = 0.0056; vehicle vs trazodone*: U* = 8, *p* = 0.0256; ***B***), total p38 (WT vs vehicle: *U* = 0, *p* = 0.0040; vehicle vs trazodone: *U* = 16, *p* = 0.2238; ***C***), and ATF4 (WT vs vehicle: *U* = 1, *p* = 0.0056; vehicle vs trazodone: *U* = 5, *p* = 0.0076; ***D***) in the cortices of 6-month-old rTg4510 mice after 9 weeks of treatment. ***E***, Protein quantification of the p-EIF2α/EIF2α ratio (WT vs vehicle: *U* = 7, *p* = 0.3758; vehicle vs trazodone: *U* = 15, *p* = 0.2824) in the cortices of 6-month-old rTg4510 mice after 9 weeks of treatment. ***F***, Representative immunoblots of P38 MAPK, ATF4, and p-EIF2α in cortical samples of WT and rTg4510 mice after 16 weeks of treatment. ***G–J***, Protein quantification of p-p38 (*U* = 14, *p* = 0.1206; ***G***), total p38 (*U* = 23, *p* > 0.9999; ***H***), ATF4 (*U* = 17, *p* = 0.3829; ***I***), and p-EIF2α/EIF2α (*U* = 18, *p* = 0.4557; ***J***) in the cortices of 8-month-old rTg4510 mice after 16 weeks of treatment. Nine week treatment: *n*(WT) = 4, *n*(vehicle) = 9, or *n*(trazodone) = 6; 16 week treatment: *n*(vehicle) = 7 or *n*(trazodone) = 7; three to eight technical replicates per sample. Immunoblot data were analyzed using the Mann–Whitney test (two tailed). Values shown are the band intensity of each protein divided by the intensity of GAPDH, with the results expressed as the respective median relative protein expression level ± minimum/maximum. ^n.s.^*p* > 0.05, *p < 0.05, #*p* < 0.01, $*p* < 0.001.

Previous studies showed that p38 MAPK, a signaling pathway recruited by microglial activation and the UPR, is inhibited by trazodone *in vitro* under inflammatory conditions (Daniele et al., 2015). We sought to confirm the effects of trazodone on p38 MAPK protein expression. Phosphorylated p38 MAPK (Fig. 2*A*,*B*; *U* = 1, *p* = 0.0056, Mann–Whitney *U* test) and endogenous total levels of p38 MAPK (Fig. 2*A*,*C*; *U* = 0, *p* = 0.0040, Mann–Whitney *U* test) were elevated in the cortices of 6-month-old rTg4510 mice compared with age-matched WT mice, which is consistent with previous findings (Maphis et al., 2015; Bennett et al., 2018). After 9 weeks of treatment, trazodone normalized phosphorylated p38 MAPK expression (Fig. 2*A*,*B*; *U* = 8, *p* = 0.0256, Mann–Whitney *U* test), while total p38 MAPK levels remained unchanged (Fig. 2*A*,*C*; *U* = 16, *p* = 0.2238, Mann–Whitney *U* test).

As p38 MAPK signaling acts both as an effector and modulator of the UPR, we next determined ATF4 protein levels. While Western blotting showed no detectable ATF4 in WT mice, increased expression was observed in the cortex of 6-month-old rTg4510 mice (Fig. 2*D*; *U* = 1, *p* = 0.0056, Mann–Whitney *U* test). Compared with the vehicle-treated group, trazodone reduced cortical ATF4 levels (Fig. 2*D*; *U* = 5, *p* = 0.0076, Mann–Whitney *U* test) to levels similar to those observed in WT controls. Trazodone-induced inhibition of ATF4 expression was not paralleled by changes in its upstream UPR-dependent regulators—phosphorylated eukaryotic initiation factor 2 alpha (eIF2α) and total eIF2α protein levels (Fig. 2*E*; *U* = 15, *p* = 0.2824, Mann–Whitney *U* test). These data suggest that the effect of trazodone on ATF4 is independent of upstream PERK/eIF2α activation and might result from alternative modulators, such as p38 MAPK signaling processes (Jiang et al., 2013). After 16 weeks of treatment, phosphorylated p38 MAPK (Fig. 2*F*,*G*; *U* = 14, *p* = 0.1206, Mann–Whitney *U* test), constitutive p38 MAPK (Fig. 2*H*; *U* = 23, *p* = 0.9015, Mann–Whitney *U* test), ATF4 (Fig. 2*I*; *U* = 17, *p* = 0.3829, Mann–Whitney *U* test), and phosphorylated eIF2α and total eIF2α (Fig. 2*J*; *U* = 18, *p* = 0.4557, Mann–Whitney *U* test) protein levels showed no statistical differences between the trazodone- and vehicle-treated groups.

**Figure 3.**
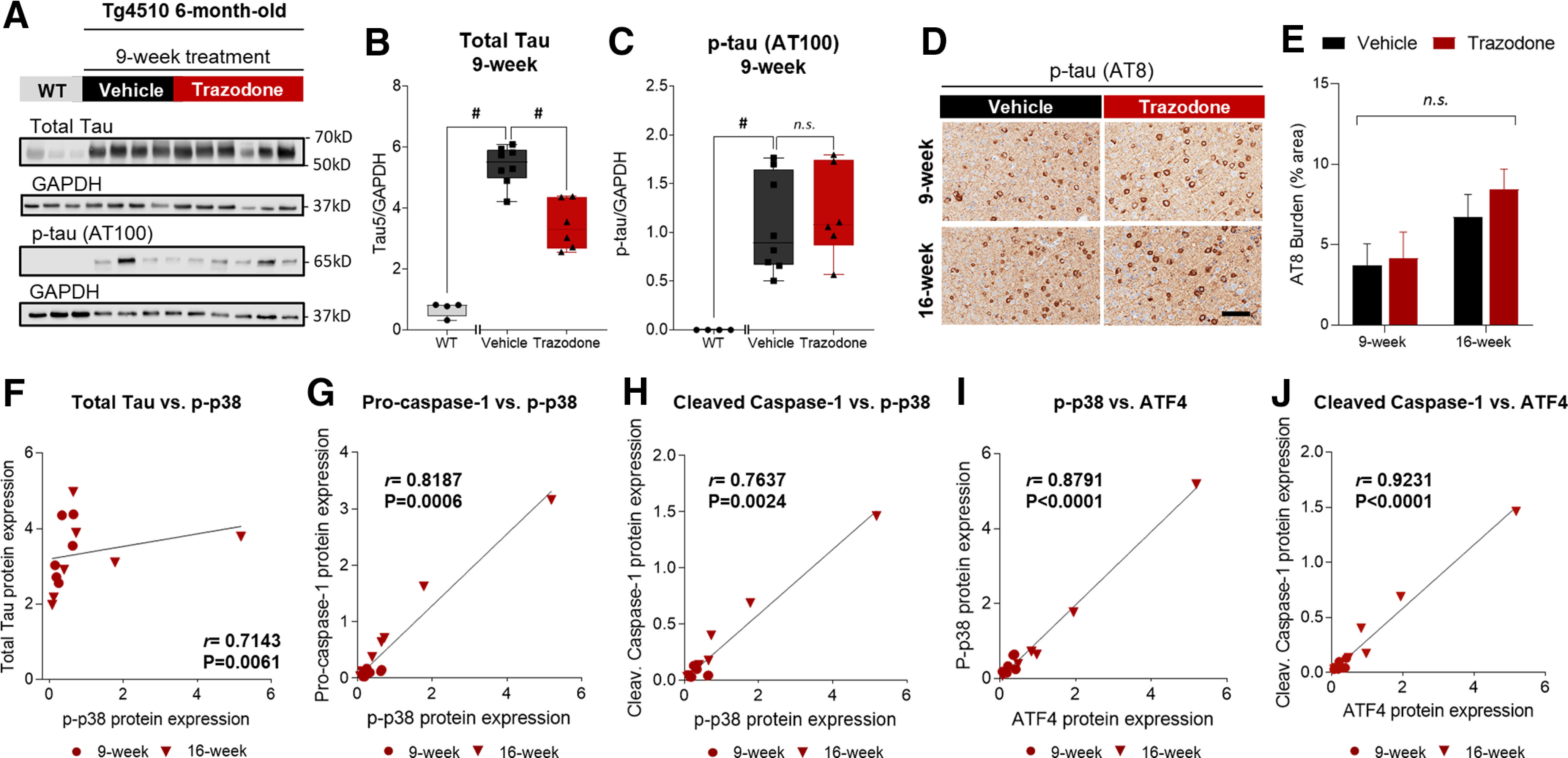
Trazodone reduces total tau but not phosphorylated tau levels in the cortices of rTg4510 mice. ***A***, Total tau (tau5) and p-tau (AT100) immunoblots of cortical rTg4510 samples collected after 9 weeks of treatment. ***B***, Total tau protein levels in cortical extracts after 9 weeks of treatment (WT vs vehicle: *U* = 0, *p* = 0.0040; vehicle vs trazodone: *U* = 2, *p* = 0.0027). ***C***, Phospho-tau protein levels in cortical extracts after 9 weeks of treatment (WT vs vehicle: *U* = 0, *p* = 0.0040; vehicle vs trazodone: *U* = 17, *p* = 0.4136). *n*(WT) = 4, *n*(vehicle) = 8–9, or *n*(trazodone) = 6; three to eight technical replicates per sample. Immunoblot data were analyzed using the Mann–Whitney test (two tailed). Values shown in graphs are the band intensity of each protein divided by the intensity of GAPDH, with the results expressed as the respective median relative protein expression level ± minimum/maximum. ***D***, Representative cortical sections of trazodone- or vehicle-treated rTg4510 mice immunostained with AT8 antibody (p-tau). Scale bar, 50 µm. ***E***, AT8 p-tau burden in the cortex of trazodone-treated rTg4510 mice after 9 and 16 weeks of treatment. *n*(vehicle) = 8–9, *n*(trazodone) = 6–10. Data are represented as LSMean ± 95% CI. Data analyzed using a general linear mixed model with repeated measures (SAS version 9.4). ***F–J***, Positive Spearman's correlation between p-p38 protein levels and total tau (***F***), pro-caspase-1 (***G***), cleaved caspase-1 (***H***), and ATF4 (***I***), as well as between ATF4 and cleaved caspase-1 (***J***) in the cortex of trazodone-treated rTg4510 mice. Correlations performed by comparing the effect after 9 weeks (*n* = 6, filled circles) and after 16 weeks of treatment (*n* = 7, inverted triangles). Simple linear regression was computed using GraphPad Prism (version 9.2.0). ^n.s.^*p* > 0.05, *p < 0.05, #*p* < 0.01, $*p* < 0.001.

We next focused on total tau and phosphorylated tau^Thr212, Ser214^ protein levels, using tau-5 and AT100 antibodies, respectively (Fig. 3*A*). In accordance with previous publications (Ramsden et al., 2005; Santacruz et al., 2005), tau burden was increased in 6-month-old rTg4510 mice with enhanced expression of total tau and phosphorylated tau^Thr212,Ser214^ (Fig. 3*B*,*C*). Nine weeks of trazodone treatment significantly reduced total tau levels compared with the vehicle control group (Fig. 3*B*; *U* = 2, *p* = 0.0027, Mann–Whitney *U* test), but not phosphorylated tau^Thr212,Ser214^ levels (Fig. 3*C*; *U* = 17, *p* = 0.4136, Mann–Whitney *U* test). The lack of an effect of trazodone on phosphorylated tau was confirmed by immunohistochemistry using the anti-AT8 antibody directed against phosphorylated tau at Ser202/Thr205 (Fig. 3*D*,*E*; treatment: *F*_(1,29)_ = 2.39, *p* = 0.1329; treatment × week interaction: *F*_(1,29)_ = 0.87, *p* = 0.3598; general linear mixed model). No significant effects of trazodone on total tau and phospho-tau^Ser202/Thr205^ (Fig. 3*E*) were observed over 16 weeks of treatment.

Together, these data indicate that trazodone can transiently reduce or even normalize the expression of several cellular components involved in tau pathophysiological processes (i.e., microglial NLRP3 inflammasome activation, phosphorylated p38 MAPK, and ATF4 expression), paralleled by a reduction in total tau levels. Preclinical evidence suggests a putative link between p38 MAPK and a PERK/eIF2α-independent activation of ATF4 (Jiang et al., 2013), and the latter has been shown to instigate NLRP3 inflammasome activation *in vivo* (T. Li et al., 2020). In accordance with this, we found that after 9 weeks of treatment with trazodone, the protein levels of phosphorylated p38 MAPK were positively correlated with the levels of total tau (Fig. 3*F*; *r* = 0.7143, *p* = 0.0061, Spearman's correlation), as well as with the NLRP3 pro- and cleaved-caspase 1 (Fig. 3*G*: *r* = 0.8187, *p* = 0.0006; Fig. 3*H*: *r* = 0.7637, *p* = 0.0024; Spearman's correlation) and ATF4 (Fig. 3*I*; *r* = 0.8791, *p* < 0.0001, Spearman's correlation), while ATF4 levels positively correlated with cleaved-caspase 1 (Fig. 3*J*; *r* = 0.9231, *p* < 0.0001, Spearman's correlation). These observations suggest that the p38 MAPK signaling pathway mediates the cross talk between the NLRP3 inflammasome and the UPR effector ATF4 to modulate total tau levels.

### Chronic trazodone treatment selectively enhanced faster EEG oscillations and REM sleep duration in rTg4510 mice

**Figure 4.**
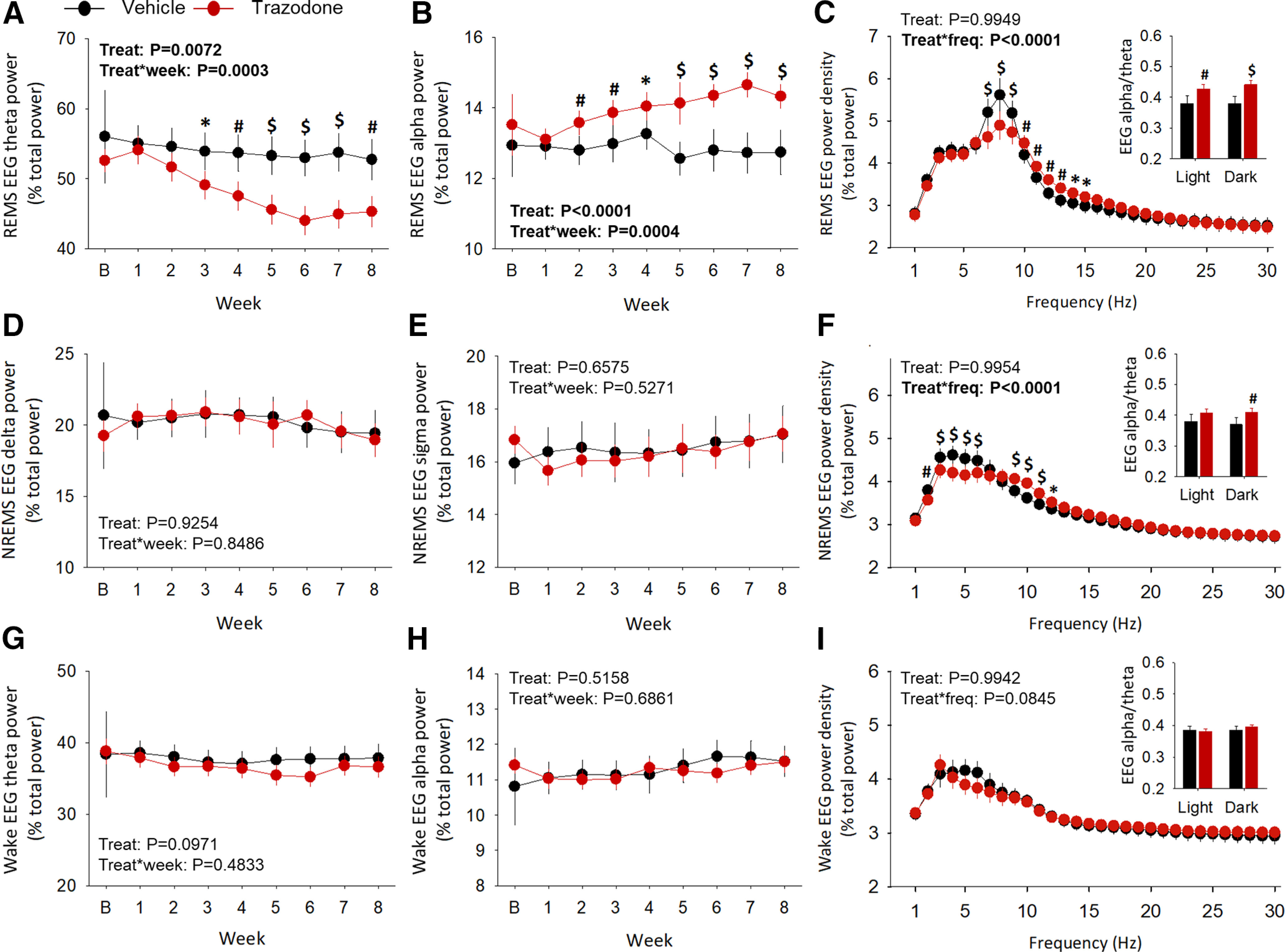
Trazodone reduced theta oscillations in REM sleep and enhanced REM sleep duration in rTg4510 mice. ***A***, ***B***, Relative 24 h REMS EEG theta (***A***; 4–8.9 Hz) and EEG alpha (***B***; 9–11.9 Hz) power density over 8 weeks of treatment. ***C***, Relative 24 h REMS EEG power spectra during the 8 weeks treatment period; indented bar graph: REMS EEG alpha/theta ratio power spectra in the 12 h light and 12 h dark periods (treatment: *F*_(1,8.55)_ = 19.11, *p* = 0.0020; light: *p* = 0.0053; dark: *p* = 0.0006). ***D***, ***E***, Relative 24 h NREMS EEG delta (***D***, 0.5–3.9 Hz) and sigma (***E***, 10–14.9 Hz) power density over 8 weeks of treatment. ***F***, Relative 24 h NREMS EEG power spectra over 8 weeks of treatment; indented bar graph: NREMS EEG alpha/theta ratio power spectra in the 12 h light and 12 h dark periods (treatment: *F*_(1,8.37)_ = 8.32, *p* = 0.0194; light: *p* = 0.0615; dark: *p* = 0.0072). ***G***, ***H***, Relative 24 h wake EEG theta (***G***; 4–8.9 Hz) and EEG alpha activity (***H***; 9–11.9 Hz) power density over 8 weeks of treatment. ***I***, Relative 24 h wake EEG power spectra during 8 weeks of treatment; indented bar graph: wake EEG alpha/theta ratio power spectra in the 12 h light and 12 h dark periods (treatment: *F*_(1,7.76)_ = 0.18, *p* = 0.6848). *n*(vehicle) = 5–6 or *n*(trazodone) = 6–8 from baseline to 8 weeks. Relative EEG power density and EEG power spectra as a percentage of total EEG power. Data are represented as LSMean ± 95% CI. Data are analyzed using a general linear mixed model with repeated measures and baseline as covariate (SAS version 9.4). *Post hoc* comparisons for significant treatment × week or frequency interactions represented as *p < 0.05, #*p* < 0.01, $*p* < 0.001.

**Figure 5.**
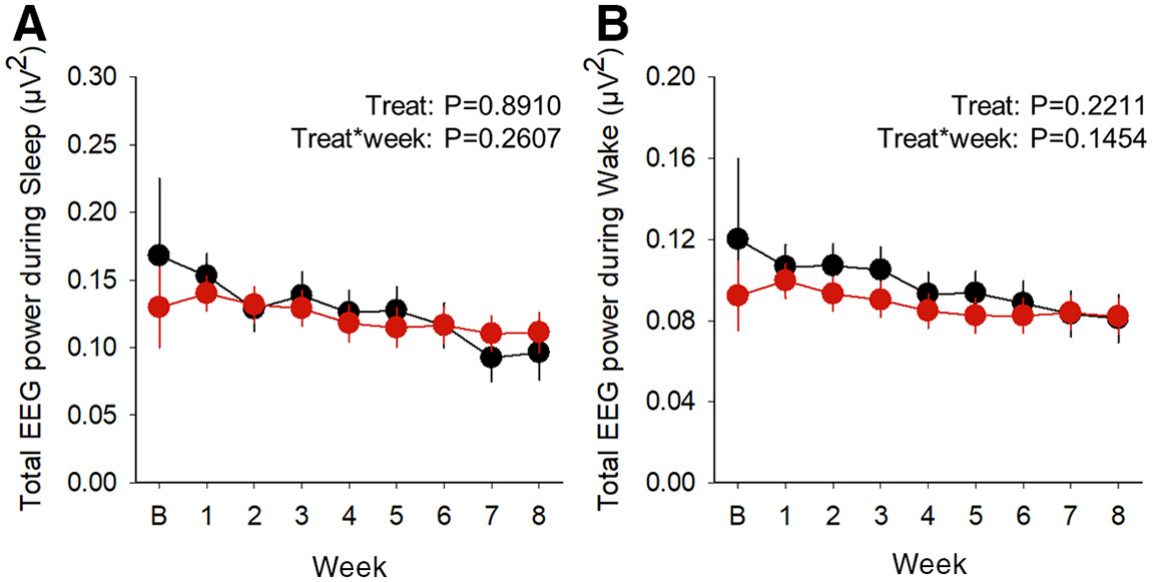
Trazodone did not affect the total EEG power during sleep or wakefulness in rTg4510 mice. ***A***, ***B***, Total EEG power during sleep (***A***) and (***B***) wakefulness over the 8 week treatment course. *n*(vehicle) = 5–6 or *n*(trazodone) = 6–8 from baseline to 8 weeks. Data are represented as LSMean ± 95% CI. Data are analyzed using a general linear mixed model with repeated measures and baseline as the covariate (SAS version 9.4).

We characterized the effects of trazodone on sleep by continuous EEG/EMG recordings over a period of 8 weeks (Fig. 1*A*, cohort 1). Quantitative EEG analysis revealed that trazodone significantly reduced relative EEG theta activity (4–8.9 Hz) during REMS from the third week of treatment (Fig. 4*A*; treatment × week interaction: *F*_(7,54.8)_ = 4.79, *p* = 0.0003, general linear mixed model), while increasing EEG alpha power (9–11.9 Hz) from 2 weeks after the start of treatment (Fig. 4*B*; treatment × week interaction: *F*_(7,54)_ = 4.66, *p* = 0.0004, general linear mixed model). Relative EEG power spectra confirmed a decrease in power between 7–9 Hz during REMS, and an increase in power in frequencies between 10 and 15 Hz, encompassing the alpha frequency range (Fig. 4*C*; treatment × frequency interaction: *F*_(29,2609)_ = 4.21, *p* < 0.0001, general linear mixed model). This effect was observed both in the 12 h light and 12 h dark phases (Fig. 4*C*, indented bar graph). By contrast, the EEG hallmarks of NREMS [i.e., EEG delta activity (0.5–3.9 Hz; Fig. 4*D*; treatment: *F*_(1,9.31)_ = 0.01, *p* = 0.9254; treatment × week interaction: *F*_(7, 52.1)_ = 0.47, *p* = 0.8486, general linear mixed model) and sigma activity (10–14.9 Hz; Fig. 4*E*; treatment: *F*_(1,5.19)_ = 0.22, *p* = 0.6575; treatment × week interaction: *F*_(7, 41)_ = 0.88, *p* = 0.5271, general linear mixed model] were not affected by trazodone over the 8 week treatment. However, relative EEG power spectra showed a reduction in NREMS EEG power between 2 and 6 Hz, and an increase between 9 and 12 Hz, compared with the vehicle group (Fig. 4*F*; treatment × frequency interaction: *F*_(29,2609)_ = 3.66, *p* < 0.0001, general linear mixed model). These changes in EEG power during NREMS were confined to the 12 h dark phase (Fig. 4*F*, indented bar graph). EEG theta activity during wakefulness showed a non significant overall decrease in trazodone-treated mice (Fig. 4*G*; treatment: *F*_(1,14.6)_ = 3.14, *p* = 0.0971, general linear mixed model), while no significant changes were observed in relative EEG alpha power (Fig. 4*H*; treatment: *F*_(1,9.02)_ = 0.46, *p* = 0.5158; treatment × week interaction: *F*_(7,54.9)_ = 0.68, *p* = 0.6861, general linear mixed model). Relative EEG power spectra confirmed a small but not significant reduction for frequencies (5–7 Hz) encompassing the theta frequency range (Fig. 4*I*; treatment × frequency interaction: *F*_(29,2609)_ = 1.38, *p* = 0.0845, general linear mixed model). Trazodone did not alter the absolute total EEG power during sleep (Fig. 5*A*; treatment: *F*_(1,12.4)_ = 0.02, *p* = 0.8910; treatment × frequency interaction: *F*_(7,58)_ = 1.31, *p* = 0.2607, general linear mixed model) and wakefulness (Fig. 5*B*; treatment: *F*_(1,12.1)_ = 1.67, *p* = 0.2211; treatment × frequency interaction: *F*_(7,65.1)_ = 1.62, *p* = 0.1454, general linear mixed model).

**Figure 6.**
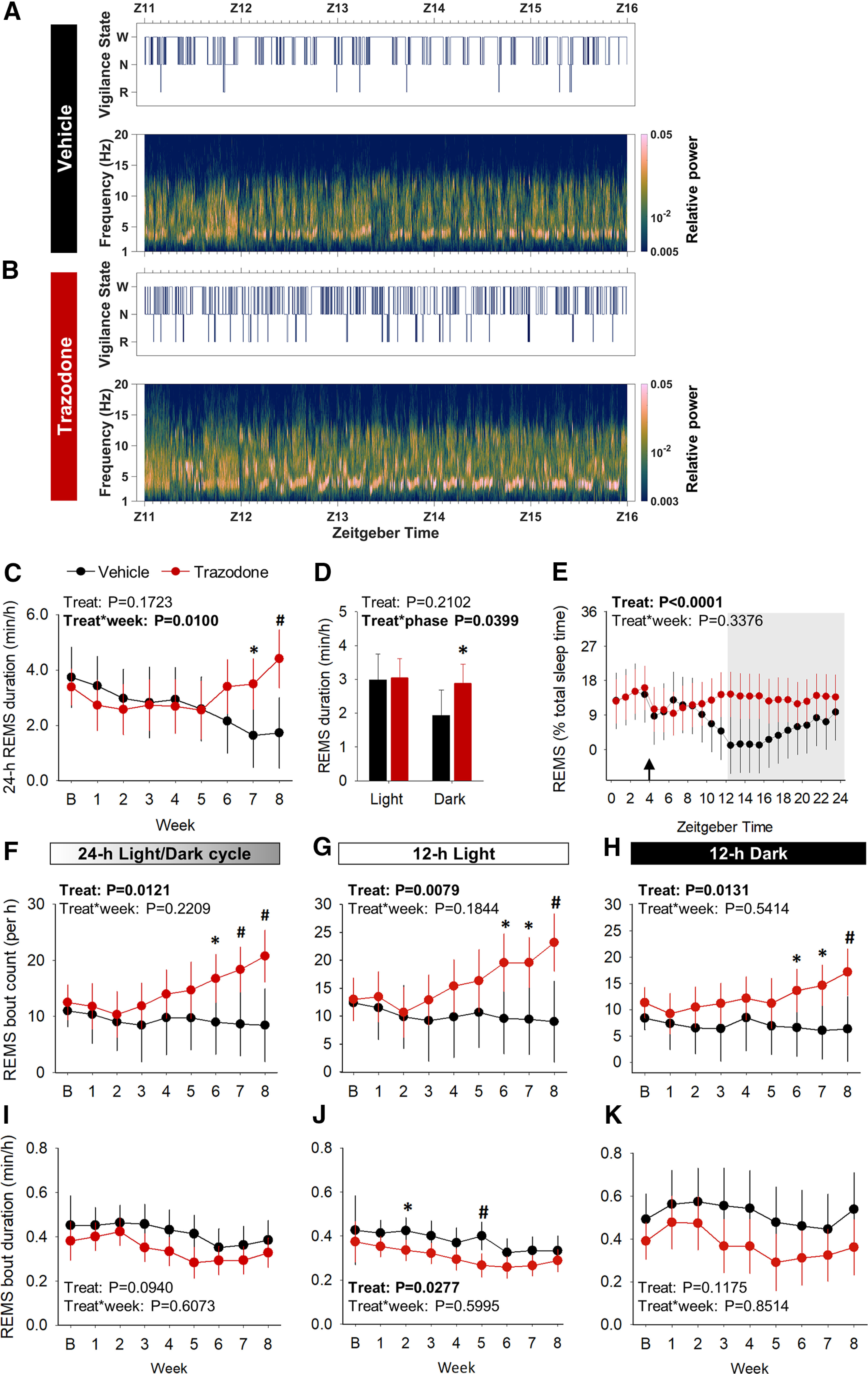
Trazodone selectively increased REMS duration after 6 weeks of treatment in rTg4510 mice. ***A***, ***B***, Representative hypnograms and spectrograms between ZT11 and ZT16 after 8 weeks of vehicle (***A***) and trazodone (***B***) treatment in rTg4510 mice. ***C***, Averaged 24 h REMS duration per hour over the 8 weekd treatment (*post hoc* analyses: 7 weeks: *p* = 0.0145; 8 weeks: *p* = 0.0019). ***D***, Average time spent in REMS over the 8 weeks treatment course during the 12 h light period (*post hoc* analyses: *p* = 0.8937) and 12 h dark period (*post hoc* analyses: *p* = 0.0414). ***E***, 24 h REMS duration as a percentage of total sleep time summed by 1 h intervals over the last 3 weeks of treatment. Arrow, time of drug administration (ZT4); gray area corresponding to the 12 h dark period. ***F***, ***I***, Averaged 24 h REMS bout count (*post hoc* analyses: 6 weeks: *p* = 0.0346; 7 weeks: *p* = 0.0074; 8 weeks: *p* = 0.0003; ***F***) and bout duration per hour over 8 weeks of trazodone treatment (***I***). ***G***, ***H***, ***J***, ***K***, Averaged 12 h REMS bout count and duration per hour during the 12 h light (*post hoc* analyses: 'count': 6 weeks: *p* = 0.0175, 7 weeks: *p* = 0.0111, 8 weeks: *p* = 0.0022; 'duration': 2 weeks: *p* = 0.0255, 5 weeks: *p* = 0.0037; ***G***, ***J***) and 12 h dark period (*post hoc* analyses: count: 6 weeks: *p* = 0.0442; 7 weeks: *p* = 0.0132; 8 weeks: *p* = 0.0060; ***H***, ***K***). *n*(vehicle) = 5–6 or *n*(trazodone) = 6–8 from baseline to 8 weeks. Data are represented as LSMean ± 95% CI and analyzed using a general linear mixed model with repeated measures and baseline as the covariate (SAS version 9.4). *Post hoc* comparisons for significant treatment × week interactions represented as *p < 0.05, #*p* < 0.01, $*p* < 0.001. B, Baseline; Treat, treatment; Z, Zeitgeber time.

In addition, trazodone also induced a selective increase in 24 h REMS duration after 6 weeks of treatment [Fig. 6*A*,*B*; treatment × week interaction: *F*_(7,70.9)_ = 2.90, *p* = 0.0100, general linear mixed model (Fig. 6*C*)]. REMS enhancement primarily occurred during the 12 h dark phase [i.e., active period for nocturnal mice; Fig. 6*A*,*B*; treatment × phase interaction: *F*_(1,191)_ = 5.75, *p* = 0.0399 (Fig. 6*D*); treatment: *F*_(1,263)_ = 26.51, *p* < 0.0001 (Fig. 6*E*); general linear mixed model]. The increase in 24 h REMS duration corresponded to an increased number of REMS episodes (Fig. 6*F*; treatment: *F*_(1,10.3)_ = 9.21, *p* = 0.0121, general linear mixed model), whereas their duration remained unchanged throughout the study (Fig. 6*I*; treatment effect: *F*_(1,8.06)_ = 3.60, *p* = 0.0940, general linear mixed model). The increased number of REMS episodes was observed both in the light and dark phases in the last 3 weeks of treatment (Fig. 6*G*,*H*).

**Figure 7.**
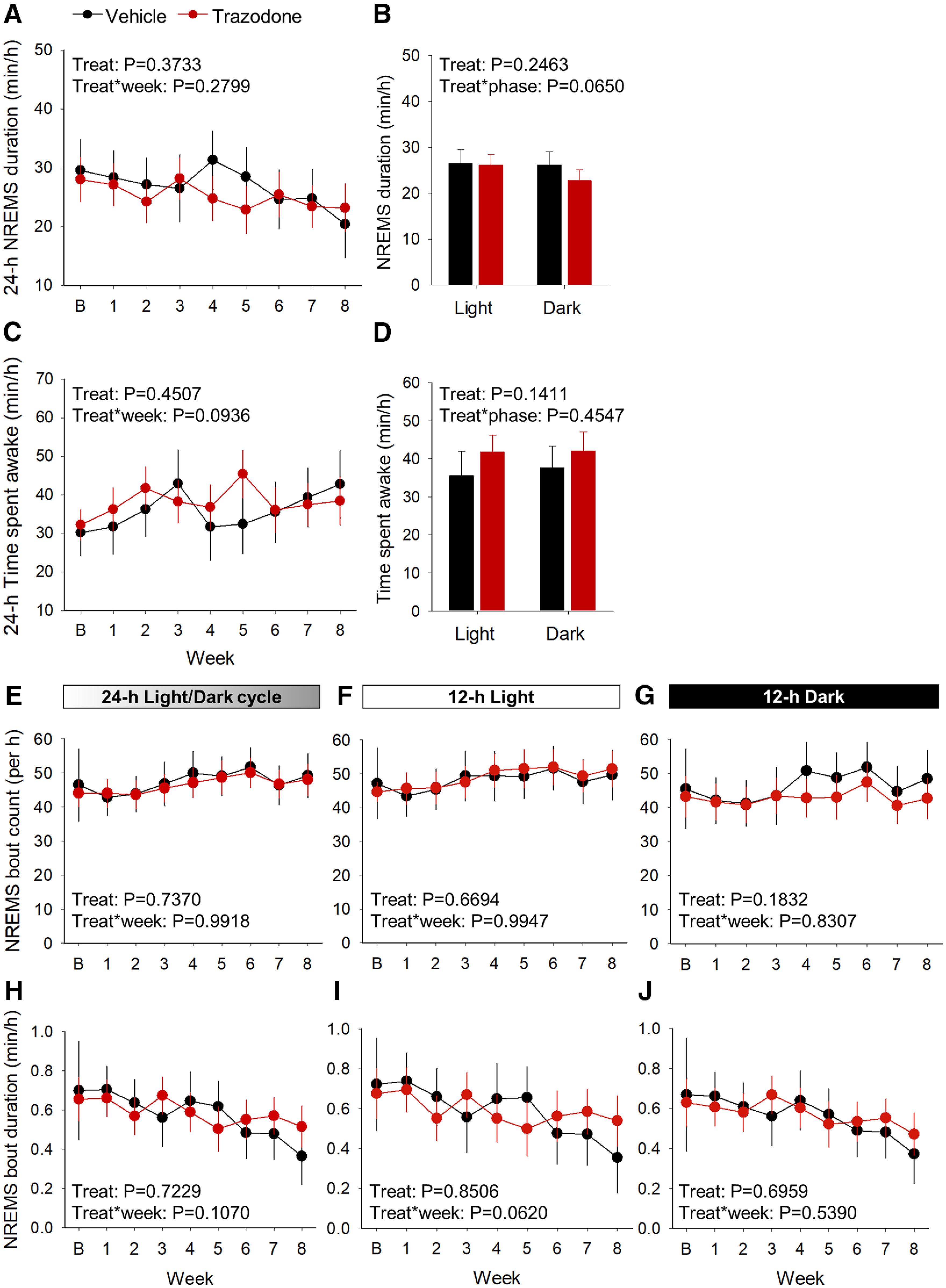
NREMS and total sleep time remain unchanged in trazodone-treated rTg4510 mice after 8 weeks of treatment. ***A***, Averaged 24 h NREMS duration per hour over 8 weeks of trazodone treatment. ***B***, Average time spent in NREMS during the 8 weeks treatment course during the 12 h light and 12 h dark periods. ***C***, Time spent awake per hour per 24 h cycle over 8 weeks of treatment. ***D***, Average time spent awake during the 8 weeks treatment course in the 12 h light and 12 h dark periods. ***E–J***, Averaged24 h NREMS bout count and bout duration per hour over 8 weeks of trazodone treatment during the 24 h cycle (***E***, ***H***) and during 12 h light (***F***, ***I***) and 12 h dark (***G***, ***J***) periods. *n*(vehicle) = 5–6 or *n*(trazodone) = 6–8 from baseline to 8 weeks. Data represented as LSMean ± 95% CI and data analyzed using a general linear mixed model with repeated measures and baseline as the covariate (SAS version 9.4). B, Baseline; Treat, treatment.

Twenty-four hour NREMS duration and 24 h total sleep time were not significantly altered by trazodone (Fig. 7*A*: treatment: *F*_(1,9.28)_ = 0.87, *p* = 0.3733; treatment × week interaction: *F*_(7,63.9)_ = 1.27, *p* = 0.2799; Fig. 7*B*: treatment: *F*_(1,10.5)_ = 1.51, *p* = 0.2463; treatment × week interaction: *F*_(1,167)_ = 3.45, *p* = 0.0650; Fig. 7*C*: treatment: *F*_(1,10.1)_ = 0.62, *p* = 0.4507; treatment × week interaction: *F*_(7,62.8)_ = 1.85, *p* = 0.0.0936; Fig. 7*D*: treatment: *F*_(1,11.9)_ = 2.48, *p* = 0.1411, treatment × week interaction: *F*_(1,171)_ = 0.56, *p* = 0.4547; general linear mixed model). NREMS continuity was not significantly affected by trazodone over the 8 week treatment (Fig. 7*E–J*).

### Trazodone transiently improved olfactory memory after 4 weeks of treatment in rTg4510 mice, while spatial working memory remained unaffected

**Figure 8.**
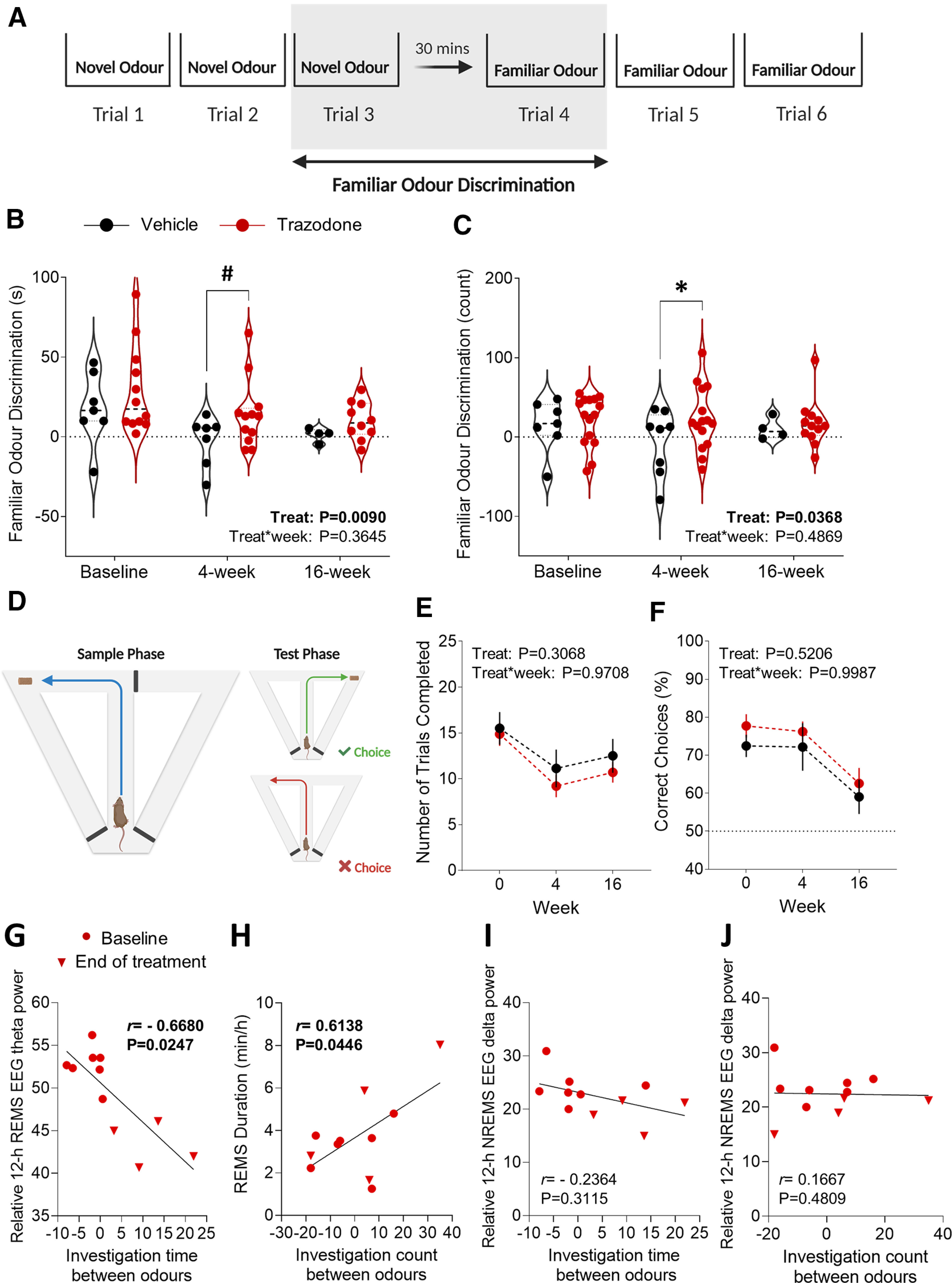
Olfactory memory was transiently improved in trazodone-treated rTg4510 mice. ***A***, Odor discrimination test protocol. Familiar odor discrimination calculated between first familiar odor presentation and last novel odor trial. ***B***, ***C***, Difference of time (***B***) and frequency (***C***) spent investigating the familiar odor versus the novel cue after 4 and 16 weeks of trazodone treatment (*post hoc* analyses: time: 4 weeks, *p* = 0.0073; 16 weeks, *p* = 0.2220; frequency: 4 weeks, *p* = 0.0224; 16 weeks, *p* = 0.3806). ***D***, T-maze rewarded alternation task protocol. ***E***, ***F***, Averaged number of trials completed (***E***) and percentage of correct choices (***F***) in the T-maze after 4 and 16 weeks of trazodone treatment. *n*(vehicle) = 4–7 or *n*(trazodone) = 11–15 from baseline to 16 weeks. Data are represented as LSMean ± 95% CI. Data are analyzed using a general linear mixed model with repeated measures and baseline as the covariate (SAS version 9.4). *Post hoc* comparisons for significant treatment × week interactions represented as *p < 0.05, #*p* < 0.01, $*p* < 0.001. ***G***, Negative Kendall's tau correlation between relative EEG theta power in REMS during the 12 h light period and familiar odor discrimination duration. ***H***, Positive Kendall's tau correlation between REMS duration in the 12 h light phase and familiar odor discrimination count. ***I***, ***J***, No significant correlation was found between the relative EEG delta power in NREMS during the 12 h light period and familiar odor discrimination duration (***I***) or count (Kendall's tau correlation; ***J***). Correlations performed comparing effects at baseline (filled circles) and at the end of the treatment (inverted triangles; baseline: *n* = 7; end: *n* = 4). Simple linear regression computed using GraphPad Prism (version 9.2.0). *p < 0.05, #*p* < 0.01, ^$^*p* < 0.001.

Olfactory memory dysfunction commonly occurs early and before other cognitive deficits in neurodegenerative diseases (Hawkes, 2003; Dintica et al., 2019), and is associated with tau pathology progression in Alzheimer's disease and frontotemporal dementia patients (Pardini et al., 2009; Lu et al., 2019). We assessed the effect of trazodone treatment on olfactory memory using an odor discrimination task. This test was conducted on parallel groups of rTg4510 mice treated with either trazodone (40 mg/kg/d) or vehicle, after 4 and 16 weeks of treatment (Fig. 1*A*, cohort 2). Mice were familiarized with an appetitive odor 24 h preceding the test, and their ability to recognize it after being introduced to a novel odor was assessed (Fig. 8*A*). Trazodone administration improved olfactory memory consolidation after 4 weeks of treatment in rTg4510 mice compared with the vehicle-treated group (Fig. 8*B*,*C*). Mixed-model analyses using baseline as a covariate showed that the time spent investigating the familiar odor (Fig. 8*B*; treatment: *F*_(1,6.07)_ = 14.29, *p* = 0.0090) and the frequency of investigation (Fig. 8*C*; treatment: *F*_(1,15)_ = 5.25, *p* = 0.0368) were increased in trazodone-treated mice after 4 weeks of treatment, which is indicative of improved memory consolidation. However, the effect was no longer observed after 16 weeks of treatment (Fig. 8*B*,*C*).

To further explore the beneficial effects of trazodone on memory, we evaluated spatial working memory shown to be impaired in rTg4510 mice (Blackmore et al., 2017), using the rewarded alternation T-maze task at baseline and after 4 and 16 weeks of treatment (Fig. 8*D*). Trazodone had no significant effect on the mean number of completed trials (Fig. 8*E*; treatment: *F*_(1,16.1)_ = 1.11, *p* = 0.3068) and the percentage of correct choices (Fig. 8*F*; treatment: *F*_(1,17.4)_ = 0.43, *p* = 0.5206). Thus, trazodone could not prevent spatial memory deficits in rTg4510 mice.

### Trazodone-induced reduction of olfactory memory deficits correlated with REMS duration and theta oscillations in rTg4510 mice

Olfactory cortical memory consolidation is sleep dependent (Barnes and Wilson, 2014). In particular, REMS has been shown to be involved in memory consolidation, and particularly emotional memory (Wassing et al., 2016). Thus, we investigated correlations between drug-induced reduction in olfactory memory deficits and changes in REMS. As behavioral testing was conducted during the light phase, correlative analyses were performed using the sleep EEG data in the same phase. Reduction in the 12 h REMS EEG theta activity significantly correlated with the investigation time in the odor discrimination task in trazodone-treated mice (Fig. 8*G*; *r* = −0.6680, *p* = 0.0247, Kendall's tau correlation). In addition, the increase in REMS duration during the 12 h light phase showed a positive correlation with the frequency of investigation (Fig. 8*H*; *r* = 0.6138, *p* = 0.0446, Kendall's tau correlation). This suggests that the trazodone-induced changes in REM sleep (i.e., decreased EEG theta activity reduction and increased REMS duration) are related to improvements in olfactory memory consolidation, as indexed by increases in both investigation time and frequency in the odor discrimination task. No significant correlation was observed between 12 h NREMS EEG delta activity and odor discrimination performance (Fig. 8*I*: investigation time: *r* = −0.2364, *p* = 0.3115; Fig. 8*J*: investigation count: *r* = 0.1667, *p* = 0.4809; Kendall's tau correlation).

## Discussion

Here we demonstrated that chronic treatment with trazodone reduced the activation of cellular components key to neuroinflammation and tau pathology in rTg4510 mice when tau aggregation is observed (Ramsden et al., 2005; Santacruz et al., 2005). Importantly, trazodone also corrected sleep disturbances and improved olfactory-dependent memory, which are reminiscent of early symptoms in patients with AD or FTD.

A novel finding was that trazodone concomitantly induced a reduction in microglial activation, indexed by decreased expression of IBA1 and NLRP3 inflammasome, and nonphosphorylated tau accumulation. Thus, trazodone may improve tau pathology via inhibition of the microglial NLRP3 inflammasome. Activated microglia contribute to tau spreading by incorporating extracellular tau aggregates (Bolós et al., 2016) that activate the NLRP3 inflammasome (Stancu et al., 2019). NLRP3 inflammasome depletion protected tauopathy mice from cortical tau accumulation (Ising et al., 2019). In the vehicle-treated group, microglial activation was downregulated as disease progresses in rTg4510 mice, corroborating previous findings (Fan et al., 2017).

We also show for the first time *in vivo* that trazodone decreased p38 MAPK activation. Trazodone acts as a serotonin transporter blocker and antagonist at several G-protein-coupled receptors (GPCRs; Settimo and Taylor, 2018). p38 MAPK is a downstream mediator of GPCRs and modulates serotonin transporters (Millan et al., 2008). Thus, the beneficial effects of trazodone on neuroinflammation are likely mediated by the observed decrease in p38 MAPK activation. p38 MAPK upregulation has also been associated with NLRP3 inflammasome activation in models of inflammation (Li et al., 2018; Zhou et al., 2019), while the inhibition of p38 MAPK activation reduced tau burden in mice without altering other MAPK-related kinases (Maphis et al., 2016).

**Figure 9.**
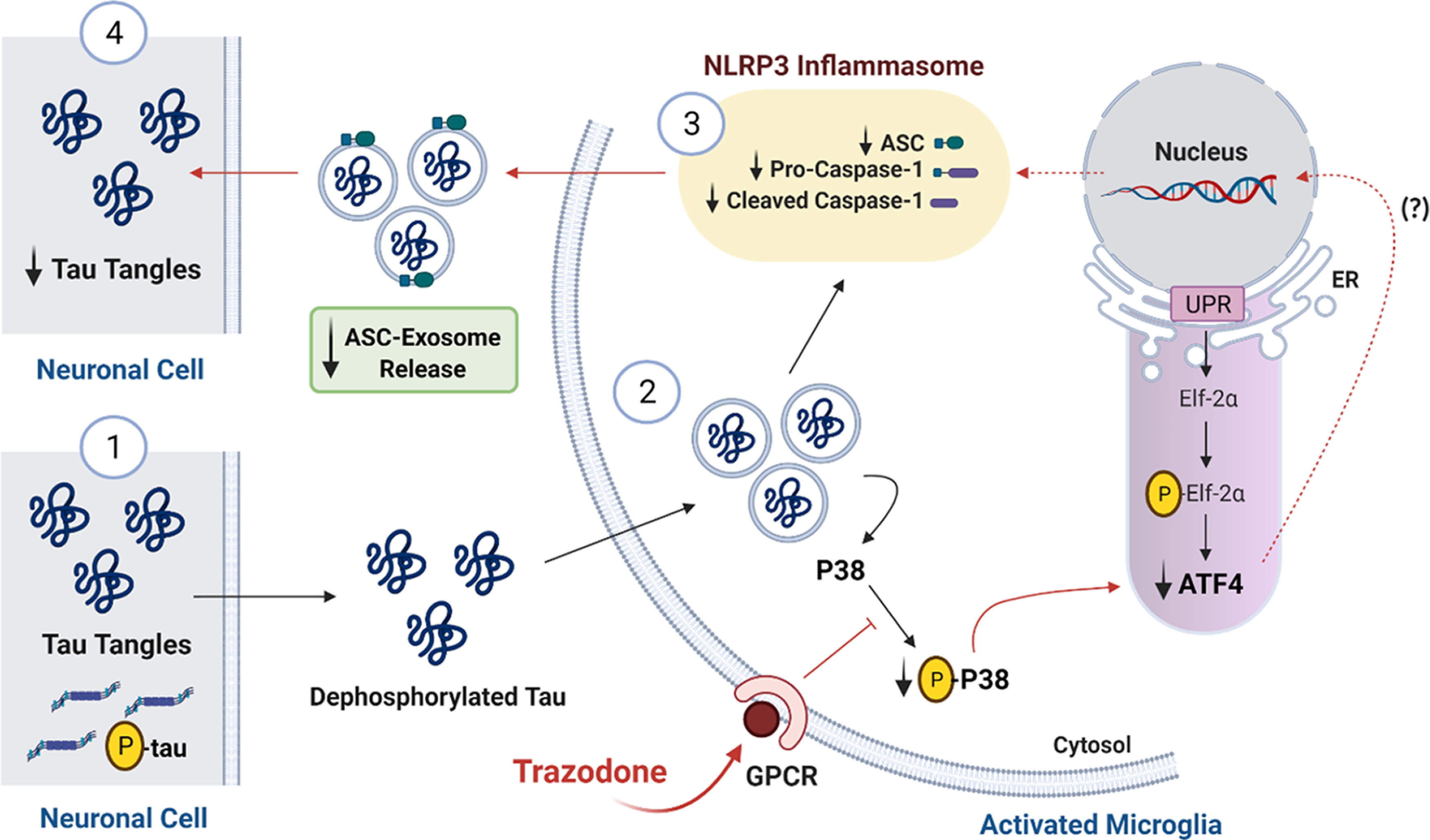
Putative mechanism of action of trazodone on microglial NLRP3 inflammasome activation and tau propagation in the brain of rTg4510 mice. Trazodone inhibits microglial activation through its GPCR antagonism (e.g., 5-HT_2A_R and H_1_R antagonism), which inhibits P38 MAPK phosphorylation. A reduction in phosphorylated p38 MAPK leads to a decrease of ATF4 expression downstream of the PERK/eIF2α UPR-dependent pathway. Decreased ATF4 expression leads to reduced expression of main NLRP3 inflammasome components (ASC, pro-caspase-1, and cleaved caspase-1). This process potentially induces a decrease in ASC-composed exosome secretion, which in turn reduces interneuronal spreading of tau protein forms, such as dephosphorylated tau. Created with BioRender. ER, Endoplasmic reticulum; p-tau, phosphorylated tau.

p38 MAPK activation promotes inflammation by activating several pathways, such as the UPR PERK/EIF2α/ATF4 pathway (Kim et al., 2020). In fact, trazodone reduced the UPR effector ATF4 in the cortex of rTg4510 mice without affecting the upstream UPR-dependent event, as shown in the hippocampus (Halliday et al., 2017). In addition, we observed a strong correlation between the expression of the NLRP3 inflammasome effector caspase-1 with phosphorylated p38 and ATF4, as well as between phosphorylated p38 and ATF4. Thus, we propose that trazodone effects are mediated by the inhibition of p38 MAPK phosphorylation through the antagonism of GPCRs expressed on microglia (Fig. 9; Haque et al., 2018). This subsequently decreases ATF4 levels downstream of its PERK/eIF2α UPR-dependent pathway. Decreased expression of phosphorylated p38 and ATF4 contributes to the downregulation of NLRP3 inflammasome components, such as ASC, pro-caspase-1, and caspase-1. We predict that this would reduce the release of ASC-dependent exosomes, which incorporate nonphosphorylated tau species and are involved in tau propagation *in vivo* (Fig. 9; Stancu et al., 2019). This putative mechanism of action is supported by the NLRP3 inflammasome activation mediated by p38 MAPK or ATF4 in inflammatory situations (Li et al., 2018; Zhou et al., 2019; W. Li et al., 2020).

These cellular pathways are implicated in the modulation of synaptic function (Freeman and Mallucci, 2016; Falcicchia et al., 2020). We therefore assessed the impact of trazodone on synaptic-related phenotypes relevant to tauopathies (i.e., sleep and memory). The current study revealed that trazodone (∼194 mg/d in humans) significantly decreased 24 h REMS theta activity, thereby reversing the pronounced 24 h EEG slowing during REMS, from 3 weeks of treatment in rTg4510 mice, which was paralleled by a smaller impact on 24 h EEG slowing in both NREMS and wake. This finding is relevant since AD patients show a pronounced slowing of EEG activity during REMS and, to a lesser extent, wakefulness (Winsky-Sommerer et al., 2019).

Reduced REMS duration is a predictor of incident dementia and one of the most prominent changes in the sleep EEG in mild cognitive impairment (Hita-Yañez et al., 2012; Pase et al., 2017). A recent longitudinal study in rTg4510 mice showed that REMS continuity was reduced with a decreased episode length in the dark and light periods from respectively 24 and 32 weeks of age, and a reduced number of episodes in the dark period from 40 weeks of age (Holton et al., 2020). A decrease in NREMS duration was also observed from 28 weeks old in the dark phase. In addition, a reduction in absolute EEG delta and theta power was observed, as well as a significant decrease in total EEG power, compared with wild-type controls (Holton et al., 2020). Decreased REMS duration and continuity, and alterations in REMS theta power, were also reported in tau (P301S) mice (Holth et al., 2017). Trazodone reversed the reduction in 24 h REMS duration and REMS continuity in rTg4510 mice in both 12 h light and 12 h dark phases. These effects are specific to REMS since trazodone did not affect 24 h total sleep time, with trazodone-treated mice displaying a tendency to spend more time awake in both phases of the 24 h cycle. Together, these results show a novel beneficial effect of trazodone on sleep parameters such as EEG slowing and REMS duration, which are altered in AD and other tauopathies.

Our work demonstrated that trazodone improved olfactory memory during the first month of treatment but did not affect spatial working memory in rTg4510 mice. Impairments in the olfactory system were previously reported in the rTg4510 mice, while olfactory deficits were observed in tau P301S mice when hippocampal-dependent spatial memory was preserved (Yang et al., 2016; Kim et al., 2017). This finding is relevant as early olfactory dysfunction is common in Alzheimer's disease (Hawkes, 2003), often occurring prior to other cognitive impairment. It is also associated with cognitive decline in elderly subjects and with tau pathology progression in AD and FTD (Pardini et al., 2009; Lu et al., 2019). In addition, we found a strong correlation between both the changes in REMS EEG theta activity and increased REMS duration, and olfactory memory decline in trazodone-treated mice. A comprehensive study in humans reported that REMS EEG slowing showed the strongest correlation with cognitive decline among sleep variables, with greater EEG slowing associated with worsening of cognitive status in AD and mild cognitive impairment (D'Atri et al., 2021). A strong relationship between resting EEG theta activity, cognitive performance, and total tau levels was also found in AD patients (Musaeus et al., 2018). While the association between REMS parameters and improved olfactory memory has not been investigated in WT mice, our findings suggest that pharmacologically improving REMS and reducing theta activity in REMS has a beneficial effect on early cognitive impairment, such as olfactory memory.

Overall, we demonstrated that a hypnotic dose of trazodone in humans (i.e., the equivalent of 194 mg/d) starting from 4 months of age, at which tau tangle-like inclusions are already present in the cortex and spatial memory is already impaired, is sufficient to transiently normalize neuroinflammation, sleep disturbances, and olfactory memory in the rTg4510 tauopathy-like mice. This provides further evidence of the potential beneficial role of trazodone for tauopathy treatment and suggests that REMS may contribute to disease progression, possibly via effects on microglial activation (Pase et al., 2017; Kaneshwaran et al., 2019; Deurveilher et al., 2021).

The transient effect of trazodone treatment observed on cellular pathology and olfactory memory may be associated with GPCR downregulation, as trazodone belongs to a class of compounds described as inducing receptor downregulation over the course of treatment (Gray and Roth, 2001), which halts their therapeutic action and often requires an increase in dose to improve treatment efficacy. Future studies should include gradual dose increments to potentially mitigate this therapeutic constraint and expand on the neuroprotective action of trazodone. Another limitation of the current work is that the long-term effect of trazodone at a hypnotic dose was investigated in the preclinical setting. Future human trials are needed to further test the effects of trazodone in patients with tauopathies. Careful consideration of prescribed dose, treatment duration, and time points when treatment starts (i.e., the prodromal, early, or advanced stage of the disease) will be important. While low hypnotic dose and short duration of treatment (50 mg and 2 weeks, respectively) showed no improvement of cognition in patients with moderate to severe AD (Camargos et al., 2015), a long-term retrospective study identified a beneficial association between trazodone and delayed cognitive decline, and this was proposed to be mediated via its effects on sleep (La et al., 2019). Future studies should also characterize the underlying mechanisms of action of trazodone in the context of tauopathy and dementia. In particular, how phosphorylated p38 MAPK and ATF4 trazodone-induced reduction mediates NLRP3 inflammasome inhibition needs to be further assessed. While not studied here, the effects of trazodone treatment on amyloid pathology and related behavioral impairments should also be investigated. A previous report showed the beneficial effect of selective 5-HT_2A_ receptor (5-HT_2A_R) antagonism against microglial dysfunction and amyloid deposition in the APP/PS1 amyloid model of AD (Lu et al., 2021).

In summary, the present study provides key insights into the putative mechanism of action underlying the beneficial effects of trazodone on cellular pathology including microglial activation. Using a sleep-promoting dose, we demonstrated that trazodone rescues EEG slowing in the rTg4510 tauopathy-like mice, and improves REMS duration and continuity, which correlated with transient olfactory memory performance. Thus, trazodone, and compounds with a similar effect on REM sleep, may be a promising treatment approach against tauopathy progression and its early symptomology.
